# Canine ehrlichiosis seropositivity and associated factors in Kenya and Tanzania: a retrospective study

**DOI:** 10.1186/s12917-023-03746-6

**Published:** 2023-09-28

**Authors:** Laboso Judy, Kihurani David, Kimeli Peter, Shah Dhaval

**Affiliations:** 1 Department of Clinical Studies, Faculty of Veterinary Medicine, University of Nairobi, P.O. Box, Nairobi, 29053- 00625 Kenya; 2 Pathologists Lancet Kenya, P. O. BOX, Nairobi, 117-00202 Kenya

**Keywords:** Canine ehrlichiosis, Serological diagnosis, SNAP 4Dx plus test, Kenya, Tanzania

## Abstract

Canine ehrlichiosis is an important tick-borne disease caused by bacteria in the Ehrlichia genus with species such as *E. canis, E. ewingii* and E. *chaffeensis* resulting in a severe dog illness. This study determined the occurrence of canine ehrlichiosis antibodies and its associated factors in Kenya and Tanzania. This was a retrospective study that evaluated laboratory records of 400 samples from Kenya and Tanzania submitted to Pathologists Lancet Kenya for the IDEXX SNAP 4Dx™ Plus test between the years 2016 and 2021. Records of all samples submitted to the Pathologists Lancet Kenya veterinary laboratory for the diagnostic tests were retrieved, examined, and compiled. Descriptive statistics and univariable and multivariable logistic regression were considered during analysis. The overall proportion of samples that tested positive for canine ehrlichiosis was 23% (92/400). Samples from Kenya accounted for 61% (245/400) of samples, and the percent positive was 31% (29/245). The samples from Tanzania accounted for 39% (155/400), and the percent positive was 69% (63/155). In the final model, the odds of a sample testing positive was 1.7 times for those submitted from July to December compared with those submitted from January to June. Blood samples of dogs from Tanzania had 5.31 times the odds of testing positive on the SNAP test when compared with those from Kenya. This study reports high percent positive in samples originating from Tanzania and those received during the year's second half.

## Background

Ehrlichiosis is an important tick-borne disease caused by bacteria in the *Ehrlichia* genus with species such as *E. canis*, *E. ewingii and E. chaffeensis,* resulting in a severe illness in dogs. *Ehrlichia spp* is a gram-negative, obligate intracellular, pleomorphic bacteria of the genus *Ehrlichia*, order Rickettsiales and family *Anaplasmataceae.* The parasite infects primarily leucocytes, forming intracytoplasmic, membrane-bound bacterial aggregates called morulae [10]. *Ehrlichia canis,* the most important species of Ehrlichia in dogs [13], is transmitted by *Rhipicephalus sanguineus,* also referred to as the brown ear tick and causes canine monocytic ehrlichiosis (CME). The disease has a worldwide distribution, but its prevalence is higher in tropical and subtropical climates [12, 14]. *Ehrlichia chaffeensis* and *E. ewingii* are transmitted by *Amblyomma americanum* and cause human infections, whereas in dogs, it results in mild to severe infections [4]. *E. ewingii* infections have only been reported in the United States because of the geographical range of the tick. However, a study by Ndip et al. [11] in Cameroon reported two dogs with *E.ewingii* DNA.

Ehrlichiosis was first reported in dogs in Nairobi, Kenya, by Danks [1] and later Murray [9], however, the causative agent was initially unknown. *Ehrlichia canis* was first confirmed in East Africa using cell culture isolation and indirect fluorescent tests [6]. The disease progression of ehrlichiosis consists of an incubation period of 8 to 10 days, followed by acute, subclinical, and in some cases, chronic phases. The disease may manifest a wide variety of clinical signs, including depression, lethargy, weight loss, anorexia, pyrexia, lymphadenomegaly, splenomegaly, pale mucous membranes due to anemia, epistaxis, petechiae, ecchymoses, prolonged bleeding during estrus, hematuria, and melena [10].

Diagnosis of canine ehrlichiosis is based on a combination of history such as tick infestation, presenting clinical signs and the specific *E. canis* tests. These include blood smear evaluation and demonstration of the typical cytoplasmic morulae in monocytes, macrophages and epithelial cells using a light microscope [5]. However, this diagnostic method has a low sensitivity as the parasites are usually found in low numbers in circulating leukocytes. A complete blood count from a dog with ehrlichiosis reveals thrombocytopenia as the most consistent finding in more than 80% of the cases, regardless of the phase of the disease [10]. Diagnosis is also based on serology, and the two methods used are Indirect Fluorescent Antibody (IFA) test and Enzyme-linked immunosorbent assay (ELISA). The IFA test is the golden standard for detecting and titrating anti*-E. canis* antibodies [5]. Dot-ELISA kits are available for detecting *E. canis* Immunoglobulin G, one of which is the SNAP 4Dx Plus Test, which utilises synthetic peptides derived from the major immunodominant *E. canis* proteins P30 and P30-1. The reaction spots are read in the result window to determine the test result. A blue colour develops in the sample spots, indicating *E. canis* antibodies in the sample.

A study by Mbugua et al. [8] showed a declining prevalence of Ehrlichiosis in Nairobi, Kenya, although the diagnosis was based on clinical signs and blood smear evaluation, which could be subjective. The study determined the occurrence of canine ehrlichiosis antibodies and its associated factors in Kenya and Tanzania.

## Methodology

### Source of data

The study included all SNAP 4Dx Plus Test screening records of blood samples for 400 dogs from Kenya and Tanzania. The data was obtained from the Pathologists Lancet Kenya Veterinary Laboratory for the period between the years 2016 and 2021. Records of all samples from Kenya and Tanzania submitted to the Pathologists Lancet Kenya for the canine ehrlichiosis test were retrieved and compiled for analysis. Where more than one test result appeared from the same patient, only the initial test was considered for this study.

### Laboratory processing of blood sample

Blood samples from dogs presented in various clinics in Kenya and Tanzania that showed clinical signs consistent with ehrlichiosis were collected from the cephalic vein into yellow top vacutainer tubes. The blood samples were transported to Pathologists Lancet Kenya in a cool box with ice gel packs. Processing of the samples involved centrifugation in obtaining serum. The serum was collected using an automated micropipette and tested serologically.

Serological diagnosis of canine ehrlichiosis at the Pathologists Lancet Kenya was done using the SNAP 4Dx Plus test, which detects Ehrlichia antibodies. The principle behind the test is that the target analyte is mixed with a conjugate with a specific binding site. Once the test is activated, the conjugate-sample mix is spread over the control and test sites. The positive control spot has binding sites for the conjugate. The negative control spot has no binding sites for conjugate or analyte. The sample site has binding sites for the analyte. If the analyte is present, it will bind to the conjugate on the sample spot. The substrate that eventually flows over the sample will then be metabolised into a blue-coloured compound that will show physically as a blue spot on the result window. This indicates that the sample is positive for Ehrlichia, and if it's negative, there's no coloured spot formation in the result window.

### Data analysis

Data were first entered into M.S. Excel (Microsoft Inc., Sacramento, California, USA) and then imported to Stata 15.1 (StataCorp LLC, College Station, Texas, USA) for analyses. Initially, the data were checked for accuracy, coded, and analysed using descriptive statistics. Proportions were determined for both outcome and explanatory variables.

Univariable analysis using simple logistic regression was performed to determine unconditional associations with those that tested positive for the SNAP 4Dx Plus Test. Univariable associations with *p* ≤ 0.2 were eligible for multivariable analysis. Multivariable logistic regression was performed to determine factors associated with the positive SNAP 4Dx Plus Test while controlling for possible confounding among model variables. The final model was built using backward stepwise elimination, leaving those variables with a *p*-value ≤ 0.05. The area under the curve (AUC) of the receiver operating characteristic (ROC) was used to evaluate overall model performance.

## Results

### Descriptive statistics

A summary of all the variables considered in this study is shown in Table 1. Of the 400 blood samples submitted for laboratory diagnosis from 2016 to 2021, about 23% (92/400) tested positive on SNAP 4Dx Plus Test. The largest percentage of samples were received from Kenya (61%), with a percent positive of 31.5%. However, Tanzania, which submitted fewer samples, had a higher percent positive of 68.5%.

**Table 1:** Descriptive statistics and significance levels of differences in proportions of SNAP 4Dx Plus positive dogs by categories for 400 samples submitted for investigation

Variable	Category	Number (%)	Proportion positive on SNAP (%)	*P*-value
Country of sample origin	Kenya	245 (61.3)	29 (11.8)	< 0.001
	Tanzania	155 (38.7)	63 (40.7)	
Half year of sampling and testing	January to June	231 (57.8)	46 (19.9)	0.087
	July to December	169 (42.3)	46 (27.2)	
Sex of the dog	Female	148 (37.0)	36 (24.3)	0.149*
	Male	191 (47.8)	37 (19.4)	
	Unknown	61 (15.3)	19 (31.1)	

^*^Overall *p*-value

When the year the samples were submitted was considered, the highest occurrence of canine ehrlichiosis was in the year 2018 (24.5%), while the lowest (9.8%) was in the year 2020. Most samples were submitted to the laboratory in the first half of each year. There was no difference in the percent positive in either half of the year.

About 37% and 48% of the submitted samples were from female and male dogs, respectively. However, about 15% had no information on the sex of the dog from which the blood sample was collected. The proportion of declared male and female dogs that tested positive was 40% and 39%, respectively.

### Factors associated with SNAP positive test

Results from the univariable logistic regression analyses with SNAP 4Dx Plus Test, a binary outcome variable (1/0 for positive/negative), are presented in Table 1. The following variables met the *p*-value ≤ 0.2 inclusion criterion for the multivariable analyses: Sex of the dog, Half year of sampling and testing, and Country of sample origin. In the final model (Table 2), while controlling for confounding variables, the odds of a sample testing positive was 1.7 times for those submitted in July to December compared with those submitted in January to June. Blood samples of dogs from Tanzania had 5.31 times the odds of testing positive on the SNAP test compared with those from Kenya. The area under the receiver operating characteristic curve for this final model was 0.73, indicating a good overall goodness–of–fit of the observed data.

**Table 2:** Final multivariable logistic regression analysis results for SNAP positive samples belonging to 400 dogs

Variable	Category	Odd Ratio	95%CI	*P*-value
Half year of sampling and testing	January to June	Baseline		0.038
	July to December	1.70	1.02, 2.80	
Country of sample origin	Kenya	Baseline		< 0.001
	Tanzania	5.31	3.19, 8.83	

## Discussion

The study reports that the country-level proportion of canine ehrlichiosis was 31.5% and 68.5% for Kenya and Tanzania, respectively. This shows that the occurrence of Canine ehrlichiosis was higher in Tanzania than in Kenya. A previous study in Kenya reported a 1.41% median prevalence rate for 24 years [8]. In Zimbabwe, while using the Immuno Comb® Canine Ehrlichia Antibody Test Kit (Biogal-Galed Laboratories, Israel), 75.2% of sera samples tested were positive for *Ehrlichia spp.* antibodies [3]. The prevalence of *E. canis* is largely dependent on the distribution of the vector *R. sanguineus*, which occurs mainly in tropical and subtropical regions. However, as supported by Mbugua et al. [8], differences in disease prevalence in the two countries during this study could be attributed to several factors, such as the immunity of the dog populations, management practices and disruption of tick habitats by new buildings and infrastructure. Higher disease occurrence is also attributed to increased dog contact [2]. This is because the transmission of ehrlichiosis by the tick vector requires an infected dog, so increased contact between the dogs increases the chances of exposure to a tick vector infected with *E. canis*. Also, since the veterinarians sampled the dogs for the test on suspicion of having canine ehrlichiosis, the Tanzanian veterinarians might have had a more accurate tentative diagnosis.

It is important to note that most of the samples tested from Kenya were from Nairobi, where the tick vector is not present endemically, leading to the possibility of low numbers of infected patients. Additionally, some of the samples that tested positive from Kenya may have had a history of travel to areas where the vector is endemic. On the contrary, in Tanzania, all the samples tested were from Dar-es-salaam, which is known to have high populations of ticks.

Blood smear examination remains a useful diagnostic tool for clinical ehrlichiosis in dogs, and microscopy evaluation remains the most straightforward and accessible diagnostic test for most laboratories [3]. Although microscopy is highly specific and can be used to diagnose canine ehrlichiosis, varying hemoparasitaemia may lead to poor to moderate sensitivity and expertise is needed to achieve efficiency (personal communication). In our study, the clinically suspected cases were tested using the SNAP 4Dx Plus test, which is a serological test that is highly sensitive and specific and it checks for antibodies against *Ehrlichia canis* and *Ehrlichia ewingii* from active or past infection.

The results showed that the odds of a sample testing positive were 70% higher for those submitted in July to December than those submitted from January to June. Over the years, the weather and climate conditions vary and can influence positively or negatively the transmission of arthropod-borne diseases [15]. Such weather factors may include air and water temperature, rainfall, humidity, surface water and wind. Generally, good tick control strategies in all seasons would reduce disease incidence. It is worth noting that Mbugua et al. [8] found no relationship between the prevalence of canine ehrlichiosis and annual rainfall amount.

The number of submitted samples from the male dog (48%) was higher than the female (37%). However, the percent positive in males (40%) versus females (39%) was similar. In the study by Kitaa et al. [7] in Nairobi, most dogs affected by Ehrlichia were males at 54.4%. Nevertheless, it is worth noting that a significant proportion of the dog population in our study had their sex unidentified (15%).

Limitations to this study include a selection bias since this was a retrospective study targeting canine ehrlichiosis suspect cases sampled for the SNAP 4Dx Plus test, and this may not have been representative of the proportion positive for the antibodies. Even though only animals with signs of ehrlichiosis were evaluated, the clinical and haematological disorders are common to other diseases. They do not necessarily indicate that the animal has canine ehrlichiosis and could be antibodies from a past infection. Also, the sample distribution in this study was unequal since fewer clinics submitted samples from Tanzania than Nairobi. Future studies should aim to detect *Ehrlichia spp* antigen in randomly selected dogs.

In conclusion, the study reports a high percent positive in samples originating from Tanzania and those received during the second half of the year. The difference in the occurrence of canine ehrlichiosis in Kenya and Tanzania could be attributed to the different management practices, climatic factors, increased contact between dogs and he vector distribution.

## Data Availability

The datasets used and/or analysed during the current study are available from the corresponding author on reasonable request.
